# A pilot study to promote mental health by developing emotional awareness and regulation in college students

**DOI:** 10.1192/j.eurpsy.2025.1999

**Published:** 2025-08-26

**Authors:** J. T. B. D. Aguiar, M. J. Brito, A. P. Amaral

**Affiliations:** 1 Faculdade Ibiapaba; 2 Centro Integrado de Atenção à Saúde de Tianguá- CE, Tianguá, Brazil; 3 Serviço de Psiquiatria, Unidade Local de Saúde de Coimbra; 4 Instituto de Psicologia Médica, Faculdade de Medicina da Universidade de Coimbra; 5 Escola Superior de Tecnologia da Saúde de Coimbra, Instituto Politécnico de Coimbra, Coimbra; 6 Centro de Investigação e Inovação em Educação (inED), Politécnico do Porto, Porto, Portugal

## Abstract

**Introduction:**

Emotional awareness and emotion regulation are essential skills for psychological well-being, especially in college students, who frequently experience stressful situations and emotional challenges. Therefore, interventions aimed at developing skills such as stress management and frustration tolerance can be important to enhance emotional awareness and regulation, contributing to the promotion of mental health in this population.

**Objectives:**

This study aimed to evaluate the effectiveness of a mental health promotion program, focusing on the development of emotional awareness and regulation in college students.

**Methods:**

This study employed a pretest-posttest design, and nonparametric tests were used. Measures:

1. Depression Anxiety and Stress Scale (DASS-21); 2) Difficulties in Emotion Regulation Scale (DERS) and an interview with six open questions to evaluated the individual perception of the impact of the intervention. Participants: nine Portuguese female college students, with an average age of 19,77 years (SD=1.48). The intervention consisted of six face-to-face sessions, lasting 60 minutes each, with theoretical and practical aspects for skill training of stress management, emotional regulation, and frustration tolerance.

**Results:**

The results suggested a decrease in levels of anxiety (*p*=.07), depression (*p*=.03), and stress (*p*=.07) after the intervention. There was also a decrease in emotional regulation difficulties for the participants (*p*=.01), notably in difficulties regarding strategies (*p*=.02), impulses (*p*=.03), and non-acceptance (*p*=.05). For the subscales of awareness, goals, and clarity, although the results showed a decrease, there was no statistical significance. It stands out from the analysis of the students’ answers to the open questions, that they developed their emotional regulation skills and they also highlighted the importance of the close supervision and support throughout the process, as well as the practical nature of the sessions.

**Conclusions:**

The results suggest that the intervention program can contribute to enhance emotion regulation and promoting mental health of college students. Suggestions for future studies would be to replicate the intervention with a larger sample and with group control.

**Disclosure of Interest:**

None Declared

